# Development of an Aerosol Dose Collection Apparatus for *In Vitro* Dissolution Measurements of Orally Inhaled Drug Products

**DOI:** 10.1208/s12248-020-0422-y

**Published:** 2020-02-13

**Authors:** Robert Price, Jagdeep Shur, William Ganley, Gonçalo Farias, Nikoletta Fotaki, Denise S. Conti, Renishkumar Delvadia, Mohammad Absar, Bhawana Saluja, Sau Lee

**Affiliations:** 1 Pharmaceutical Surface Science Research Group, Department of Pharmacy & Pharmacology, University of Bath, Bath, BA2 7AY UK; 2 Office of Research and Standards, Office of Generic Drugs, Center for Drug Evaluation and Research, Food and Drug Administration, Silver Spring, Maryland USA; 3 Present Address: Office of New Drug Products, Office of Pharmaceutical Quality, Center for Drug Evaluation and Research, Food and Drug Administration, Silver Spring, Maryland USA; 4 Present Address: Office of Clinical Pharmacology, Office of Translational Sciences, Center for Drug Evaluation and Research, Food and Drug Administration, Silver Spring, Maryland USA; 5 Office of Testing and Research, Office of Pharmaceutical Quality, Center for Drug Evaluation and Research, Food and Drug Administration, Silver Spring, Maryland USA

**Keywords:** aerosol, inhaled corticosteroids, dissolution, bioequivalence

## Abstract

The aim of the study was to develop a robust and standardized *in vitro* dissolution methodology for orally inhaled drug products (OIDPs). An aerosol dose collection (ADC) system was designed to uniformly deposit the whole impactor stage mass (ISM) over a large filter area for dissolution testing. All dissolution tests were performed under sink conditions in a sodium phosphate buffered saline solution containing 0.2%*w*/w sodium dodecyl sulphate. An adapted USP Apparatus V, Paddle over Disk (POD), was used throughout the study. The dissolution characteristics of the ISM dose of a commercial metered-dose inhaler (MDI) and a range of dry powder inhaler (DPI) formulations containing inhaled corticosteroids were tested. The uniform distribution of the validated ISM dose considerably reduced drug loading effects on the dissolution profiles for both MDI and DPI formulations. The improvement in the robustness and discriminatory capability of the technique enabled characterization of dissolution rate differences between inhaler platforms and between different DPI product strengths containing fluticasone propionate. A good correlation between *in vivo* mean absorption time and *in vitro* dissolution half-life was found for a range of the inhaled corticosteroids. The ADC system and the reproducible *in vitro* POD dissolution measurements provided a quantitative-based approach for measuring the relationship between the influence of device and the dispersion characteristics on the aerosol dissolution of low solubility compounds. The *in vitro* dissolution method could potentially be applied as a dissolution methodology for compendial, quality control release testing, and during development of both branded orally inhaled drug products and their generic counterparts.

**Electronic supplementary material:**

The online version of this article (10.1208/s12248-020-0422-y) contains supplementary material, which is available to authorized users.

## INTRODUCTION

For a locally acting inhaled drug product to elicit a pharmacological effect, the therapeutic dose must first reach the mucosal surface, lining the respiratory tract. Upon reaching the respiratory mucosa, the fate of the inhaled drug substance is not well understood. However, it is believed that the critical determinants that affect the local drug concentration at the sites of action, as well as the rate and extent of drug absorption through the lung, are the deposition pattern (i.e., the distribution of the respirable dose among mouth–throat regions, conducting and peripheral airways), the molecular properties of the active pharmaceutical ingredient (API) and the need for the drug to be in solution, that is, the *in vivo* dissolution kinetics (1).

Currently, since local drug concentrations throughout the respiratory tract cannot be measured in a practical way, determining local equivalence between test and reference products in developing bioequivalent generic products is very challenging and more complicated than for systemically acting drugs. Furthermore, with the limited understanding of the relationship among conventional *in vitro* performance parameters [e.g., aerodynamic particle size distribution (APSD) profiles, mass median aerodynamic diameter (MMAD), fine particle dose (FPD), and delivered dose] and the dissolution and absorption kinetics of the respirable dose, the development of bioequivalent orally inhaled drug products (OIDPs) is extremely challenging. The US Food and Drug Administration (FDA) currently recommend the aggregated weight of evidence approach to establish bioequivalence between test and reference OIDPs (2). This relies on comparative *in vitro* studies (for equivalence in product performance) and comparative *in vivo* studies [pharmacokinetic (PK) for equivalence in systemic exposure and pharmacodynamic (PD) or clinical endpoint (CE) for equivalence in drug delivery at the sites of action] in addition to formulation sameness (Q1 and Q2, i.e., the same inactive ingredients and at the same concentration ± 5% as the reference product) and device similarity. This weight of evidence approach has been used by FDA to draft individual product-specific guidance to assist in the development programs of generic OIDPs.

In the case of generic OIDPs, the identification, validation, and standardization of novel *in vitro* and *in silico* tools may provide an insight into the relationship between regional drug deposition and the extent and rate of drug exposure at the local sites of action in the lungs. This may ultimately provide an alternative regulatory pathway for demonstrating bioequivalence of generic OIDPs without the need to conduct *in vivo* comparative PD or CE studies.

For poorly soluble drugs, the correlation between aqueous solubility and mean absorption time (MAT) for a range of compounds in the lungs suggests that dissolution may be the rate limiting step for absorption (3). Furthermore, dissolution of the respirable dose in the limited fluid lining the central airways is in kinetic competition with the pulmonary mucociliary clearance mechanism (4). Thus, the bioavailability of the pulmonary deposited dose, both locally and systemically, may be directly affected by the dissolution characteristics in the air–liquid interface. *In silico* mechanistic modeling of the systemic exposure of a poorly soluble drug substance, with different aerodynamic particle size distributions, highlighted that the slight difference in deposition pattern could not explain the observed differences in plasma profiles and indicated that the rate of dissolution was the rate limiting step of absorption into the systemic circulation (5).

The major challenges in developing an *in vitro* dissolution test for OIDPs have been reviewed by both the USP *Ad Hoc* Inhalation Advisory Panel in 2008 and the dissolution working group of the IPAC-RS in 2012 (6,7). They reviewed all published methodologies for aerosol collection and dissolution testing of OIDPs, available at those times. The major findings of both groups were that all methodologies lacked the robustness and the level of validation required for a standardized dissolution test, either as a quality control tool to assess batch consistency or in establishing a quantitative *in vitro*–*in vivo* relationship between dissolution data and systemic PK profiles.

The major challenge in the development of a dissolution approach for OIDPs remains the aerosol collection system and the associated apparatus for measuring dissolution of a representative pulmonary dose. May *et al.* (2012) investigated the influence of different dose collection methods, membrane holders, and dissolution media on the dissolution process (8). The authors highlighted the critical need to collect a homogenous distribution of the aerosolized dose onto a membrane to increase the discriminatory capability of the dissolution measurements. Indeed, one of the drawbacks of commercially available aerosol filter-based collection systems is that the dissolution rate is highly sensitive to the collected dose and decreases with increasing collected mass of a given formulated product (8). The effect is thought to be due to the formation of *in situ* agglomerates, created by the impactor jets, during collection onto the filter membrane surface. This results in agglomerates of drug particles that cannot be fully wetted by the dissolution media. Thus, these collection systems can lead to a significant increase in the probability of particle–particle aggregation, which directly influences the sensitivity and discriminatory capability of dissolution rate measurements.

The goal of this study was to develop a bespoke aerosol dose collection (ADC) system together with an adapted USP Apparatus V, Paddle over Disk (POD) (9), which may constitute a significant step toward providing reliable dissolution data to gain a better understanding of the potential relationships among OIDP formulations and local and systemic bioavailability. The major specific, technical objective of the study was to validate the impactor stage mass (ISM) dose collected by the ADC, with respect to the ISM dose collected by the Next Generation Impactor (NGI), and to significantly improve the robustness and discriminatory capability of the *in vitro* dissolution test through uniform distribution of the aerosolized dose across a high surface area filter. All dissolution tests were undertaken with a selection of commercially available OIDPs containing glucocorticoids, which, with their poor water solubility and negligible oral bioavailability, were selected as suitable candidates for investigation.

## MATERIALS AND METHODS

### Materials

Commercial 50, 125, and 250 μg fluticasone propionate (FP) MDIs {Flixotide® Evohaler®}; 100, 250, and 500 μg FP DPIs {Flixotide® Accuhaler®}; 50/100, 50/250, and 50/500 μg salmeterol/fluticasone propionate combination (S/FP) DPIs {Seretide® Accuhaler®}; and 200/25 μg fluticasone furoate/vilanterol combination (FF/V) DPIs {Relvar® Ellipta®} were purchased from GlaxoSmithKline. Commercial 220 μg mometasone furoate (MF) DPIs {Asmanex® Twisthaler®} were purchased from Merck. All products were tested well before their expiry date.

Reference standards (1 g) of FP, FF, and MF were purchased from LGC Standards (Middlesex, UK). Sodium phosphate buffered saline (PBS) solutions {prepared using sodium phosphate monobasic dihydrate (NaH_2_PO_4_.2H_2_0, MW = 156.01 g/L), sodium phosphate dibasic (Na_2_HPO_4_, MW = 141.96 g/L), 0.1 M hydrochloric acid (HCl), and sodium chloride (NaCl, MW = 58.44 g/L)} were purchased from Fischer Scientific (Loughborough, UK). Pall A/E type glass fibre filters (47 mm diameter, 1 μm nominal pore size) were purchased from Copley Scientific (Nottingham, UK). Whatman Puradisc™ polytetrafluoroethylene (PTFE) filters (13 mm diameter, 0.2 μm pore size) were purchased from Scientific Laboratory Supplies (Nottingham, UK). Water used during the studies was Milli-Q Reverse Osmosis purified (Merck Millipore, Darmstadt, Germany). Sodium dodecyl sulphate (SDS) and polyoxyethylene (80) sorbitan monooleate (Tween 80), and methanol and acetonitrile were of high performance liquid chromatography (HPLC) grade and purchased from Sigma (Gillingham, UK). Adirondack Alcohol Ink (Raisin TIM22145) was purchased from Tim Holtz® (Ranger Ink, NJ, USA).

### Solubility Measurements

Saturated solutions of FP were prepared by adding an excess of the drug into a PBS solution at pH = 7.4 containing 0.2% *w*/*v* SDS. Solutions were held at 37°C for 24 h prior to filtration. All solubility measurements were performed in triplicate.

### Scanning Electron Microscopy (SEM)

Drug filters were sputter coated with gold (Edwards Sputter Coater S150B, Edwards High Vacuum, Sussex, UK) to achieve a thickness of approximately 20 nm. All SEM imaging was performed using a scanning electron microscope (JEOL JSM6480LV, Tokyo, Japan) using 15 kV accelerating voltage. The magnification was set to × 1000.

### Dissolution Studies

All dissolution studies were conducted in an adapted USP Apparatus V, also known as Paddle over Disk (POD), traditionally used for transdermal delivery systems (10). All dissolution measurements were performed at 37°C in a 300 mL PBS and 0.2% *w*/*v* SDS dissolution media with a stirring speed of 75 rpm. The USP disk assembly membrane holder for transdermal patches was adapted to enable a 47 mm glass fiber filter to be housed. A 50 mm diameter stainless steel disk assembly was used with a 74 mesh screen (NW-50-CR-SV-74, Nor-Cal Inc., USA). For all dissolution experiments, 3 mL aliquots were withdrawn at 2.5, 5, 10, 15, 20, 25, 30, 60, 120, 180, and 240 min time intervals and filtered with a 0.2 μm PTFE syringe filter directly into HPLC vials. To maintain a constant volume in the dissolution vessel, the sampling volume was replaced with pre-warmed dissolution media. Each dissolution study was performed in triplicate. The total amount of drug loaded onto the filters were determined by the sum of the cumulative mass released together with any mass retained on the membrane after 4 h. The fractional percentage of the drug dissolved at each time point was determined by dividing the amount of drug by the total mass loading.

### Kinetic Modeling of Drug Release and Dissolution Release Profile Comparison Testing

The dissolution profiles of all commercial products were fitted to zero-order, first-order, Higuchi, Hixson-Crowell, and Korsmeyer and Peppas models to ascertain the most appropriate kinetic modeling of drug release. For all batches of commercial products, the most appropriate model was the first-order drug release.

Model-independent methods were also applied to compare the dissolution release profiles. The mean dissolution time (MDT) of the profiles was calculated by the following equation (11):

$$ MDT=\frac{\sum_{i=1}^n{T}_i\Delta  {M}_i}{\sum_{i=1}^n\Delta  {M}_i} $$

Where *n* is the number of dissolution sample time points, *i* is the sample number, ΔMi is the fraction of drug release between *t*_i_ and *t*_(i-1)_, and *T*_i_ is the calculated midway time point between sampling, where *T*_i_ = (*t*_i_ + *t*_(i-1)_)/2.

A similarity factor (f2) analysis was also calculated to compare dissolution release profiles of MDI and DPI products containing FP between the initial sampling time point and the cumulative mass at 120 min. The f2 value was considered similar when not less than 50, which is equivalent to an average difference of no more than 10%. The similarity factor analysis has been adopted by the regulatory authorities as a criterion for the assessment of similarity between test and reference *in vitro* dissolution profiles (11).

### High Performance Liquid Chromatography (HPLC)

Chemical analyses of active pharmaceutical ingredients were detected using an HPLC system which consisted of a binary pump coupled to an autosampler and a variable wavelength UV detector (Agilent 1200, Wokingham, UK) that was set to 235 nm. The pump flow rate was set to 1.0 mL/min through a Hypersil ODS C_18_ column (Fisher Scientific, Loughborough, UK, column length of 50 mm, internal diameter of 4.6 mm, and particle size of the packing material of 5 μm), which was placed in a column oven (Agilent, Wokingham, UK) set to 40°C. The mobile phase consisted of methanol, acetonitrile, and water (32.5, 32.5, 35% *v*/v). The experimental run time was 3.5 min. For all HPLC studies, a linear regression analysis was used for the assessment of the HPLC calibration. Quantification was carried out by an external standard method, and linearity was verified between 0.05 and 50 μg/mL.

### Design and Development of the Aerosol Dose Collection (ADC) System

To overcome the apparent influence of the aerosol collection method on *in vitro* dissolution release profile, a bespoke aerosol dose collection (ADC) system was designed and built. Briefly, the main objectives were the following: (1) to collect and validate against a standard NGI the whole impactor stage mass (ISM) onto a high surface area filter, (2) to obtain dissolution release profiles independent of drug loading, and (3) to attempt to increase the overall ruggedness, reliability, and discriminatory capability of *in vitro* dissolution based measurements of OIDPs for quality control and bioequivalence testing.

**Fig. 1:**
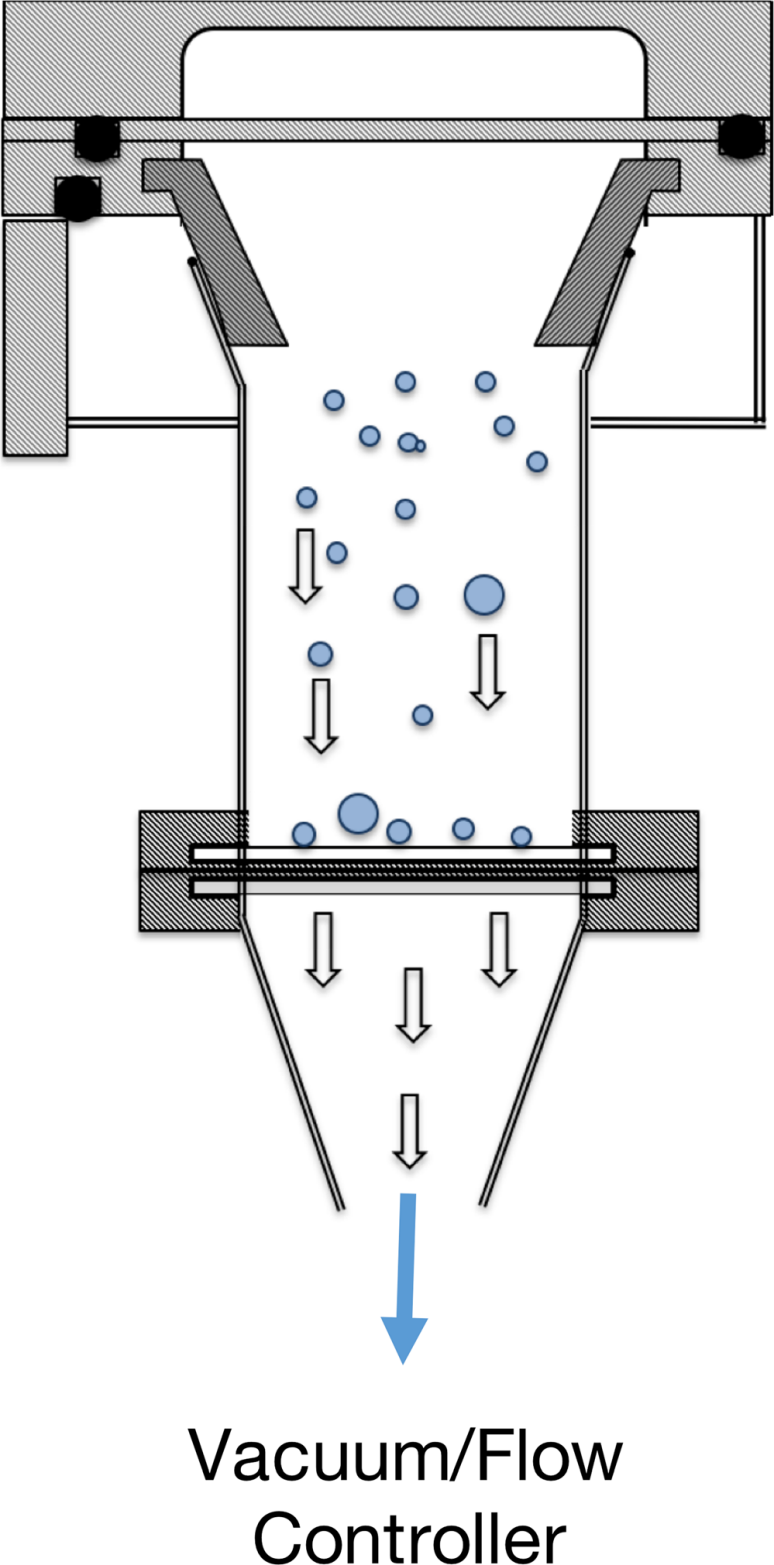
A schematic cross-sectional diagram of the aerosol dose collection (ADC) system, which was incorporated into stage 2 of the NGI

A cross-sectional schematic of the ADC system that was incorporated within stage 2 of an NGI, in this particular study, is shown in Fig. 1. The impactor nozzle in the interstage plate of the NGI was removed and replaced with a tapered, circular orifice. The removal of the jets from the impactor nozzle led to a significant reduction in the exit air velocity, while laminar flow behaviour (i.e., Reynolds number 500 < Re < 3000) was maintained across the calibrated flow rates of the NGI (30–100 L/min). The difference in the air velocity exiting stage 2 was calculated to be an order of magnitude less with the use of a single, circular orifice (from 891 cm/s with the jets to 83.7 cm/s at 60 L/min). The combination of low air flow velocity and the distribution of the whole pneumatic air across a large diameter orifice were specifically designed to enable uniform deposition of the aerosol dose.

To harvest the aerosol dose, the collection system was directly mounted onto the tapered nozzle. The dose collection housed a removable holder for an appropriate 47 mm diameter filter that was arranged orthogonally to the direction of the pneumatic flow. The dose collector was connected directly to a vacuum pump via a flow controller (TPK Model, Copley Scientific, Nottingham, UK). This enabled the collection of all the dose below any remaining NGI stage and allowed a direct unimpeded pathway extending from the orifice to the filter.

## RESULTS AND DISCUSSION

Upon initially validating the dose collection efficiency of the ADC system, the apparatus was used to investigate loaded dose effects (approximately 50–500 μg) on the dissolution release profiles of both 250 μg FP DPI and 125 μg FP MDI. The system was also used to study the relationship between mean absorption time and dissolution kinetics of a series of low solubility inhaled corticosteroids. Finally, the dissolution characteristics of FP from the three different product strengths of FP MDI, FP DPI, and S/FP DPI products were compared.

### Validation of the Dose Collection Efficiency of the ADC System

**Fig. 2:**
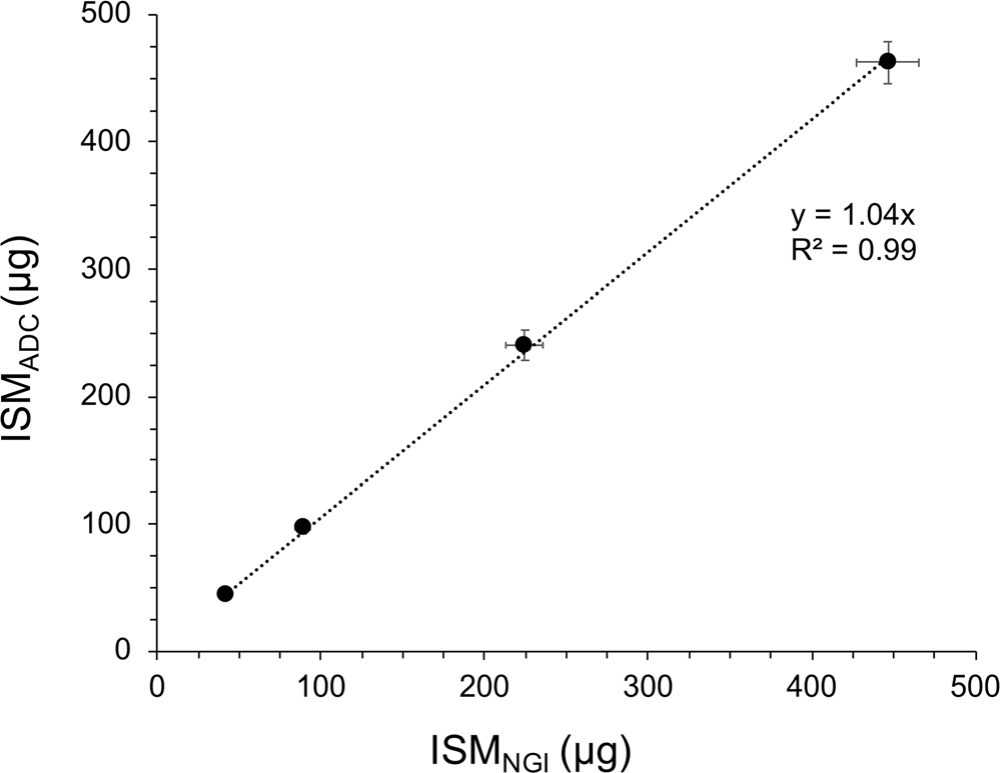
Validation of the mean impactor stage mass (ISM) collection of the ADC system (ISM_ADC_) versus the mean ISM collection of the NGI (ISM_NGI_), for increasing number of actuations of the 250 μg FP DPI tested at 60 L/min (n = 3, mean ± SD)

To validate the collection efficiency of the ADC system, the ISM collected onto the glass fiber filter within the ADC, from a commercial 250 μg FP DPI, was compared with standard *in vitro* NGI recovery at a flow rate of 60 L/min. As shown in Fig. 2, with increasing number of actuations (1, 2, 5, and 10), there is good correlation between the ISM collected from the conventional NGI and the ADC system over the range of mass loadings studied.

The uniformity of deposition across a filter surface with the ADC system was visualized by formulating an alcoholic ink (Raisin (TIM22145), Tim Holtz® Adirondack Alcohol Inks, USA) as a 0.5% *w*/*v* solution-based MDI. As shown in the photographic images in the supplementary information (Fig. S1), the uniformity and increasing intensity of the ink with increasing number of actuations suggested that the aerosol dose was being uniformly deposited across the whole collection filter surface.

**Fig. 3:**
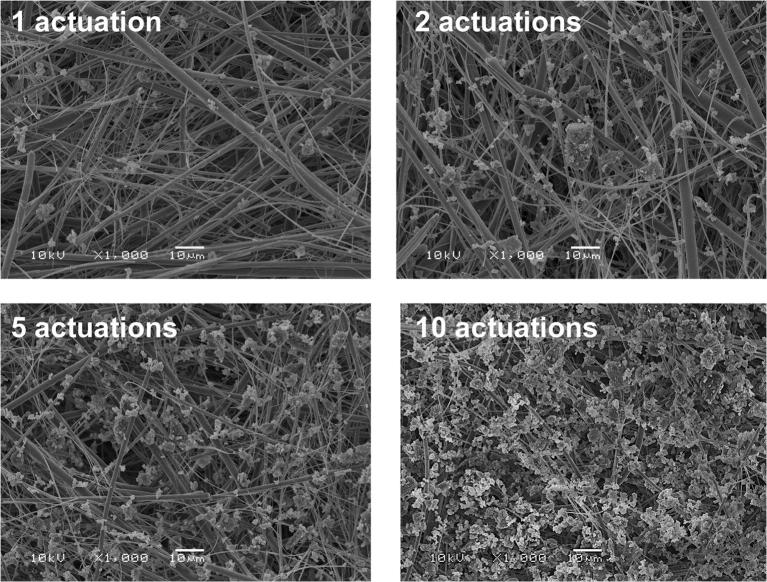
Representative scanning electron microscope (SEM) micrographs of the ISM dose collected using the ADC system for an increasing number of actuations of the 250 μg FP DPI at a flow rate of 60 L/min. Magnification × 1000 for all SEM micrographs shown

**Fig. 4:**
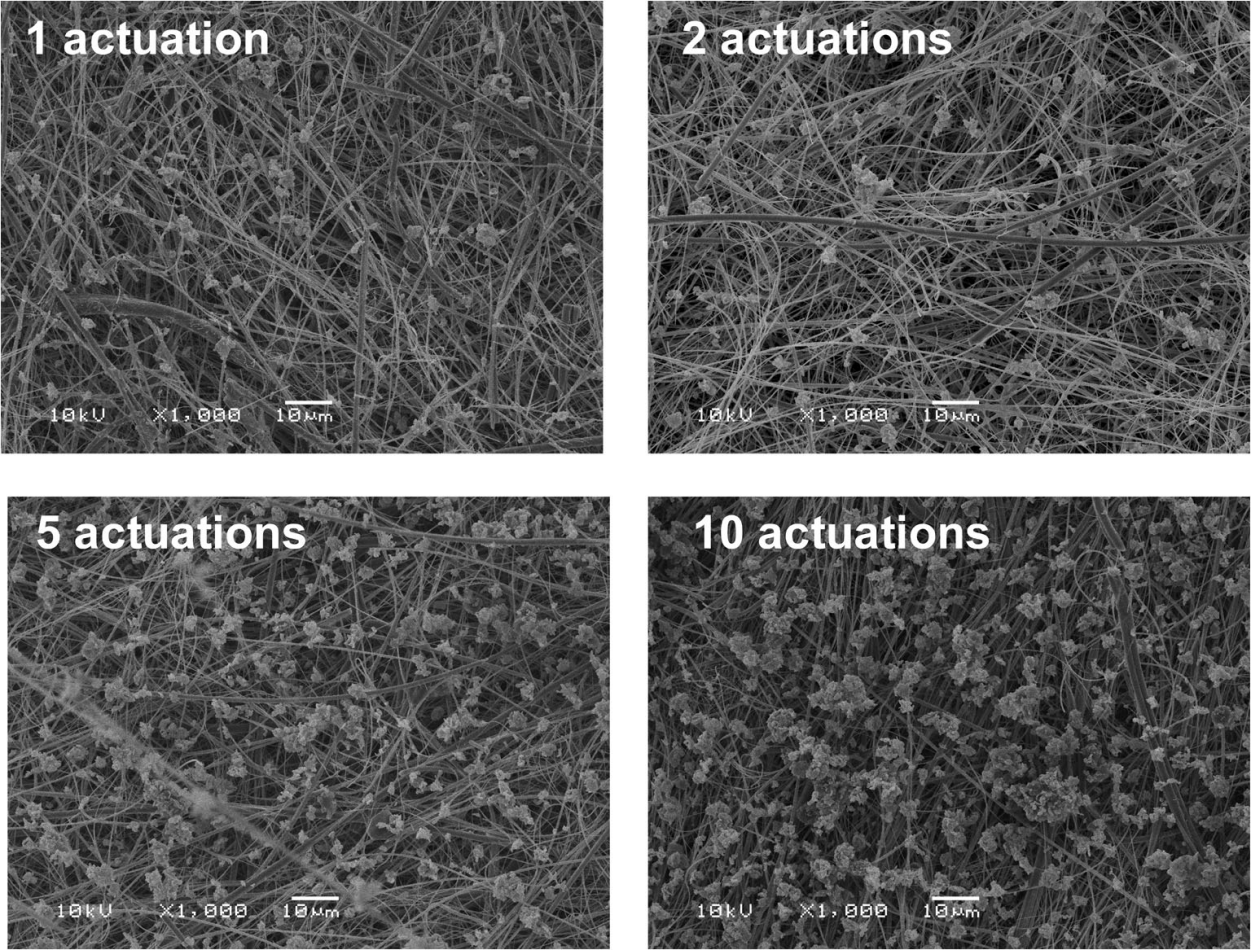
Representative scanning electron microscope (SEM) micrographs of the ISM dose collected using the ADC system for an increasing number of actuations of the 125 μg FP MDI at a flow rate of 30 L/min. Magnification × 1000 for all SEM micrographs shown

Representative scanning electron microscope (SEM) micrographs of the ISM collected dose with increasing number of actuations of the 250 μg FP DPI tested at 60 L/min and 125 μg FP MDI tested at 30 L/min are shown in Figs. 3 and 4, respectively. As indicated by the SEM micrographs, the local deposition density of the FP particles, which increased from 2.6 to 26.6 μg/cm^2^ and from 3.9 to 38.3 μg/cm^2^ for 1 and 10 actuations of the 250 μg FP DPI and 125 μg FP MDI, respectively, led to minimal aggregation and *in situ* agglomeration formation.

### Solubility Determinations of FP for Dissolution Studies

The solubility of fluticasone propionate (FP) at 37 °C in a 10 mM sodium phosphate buffered saline solution (pH = 7.4) with the addition of 0.2% *w*/w SDS was 14.2 ± 3.4 μg/mL. To ensure that sink conditions could be maintained over a wide range of FP drug loading, the concentration in the dissolution media should not exceed 10% of the saturated solubility in the respective media (10,11). The total volume of dissolution media (300 mL) was selected to maintain sink conditions with increasing drug loading while maintaining sufficient sensitivity to detect any formulation differences.

### Dissolution Release Profiles as a Function of Loaded Dose

**Fig. 5:**
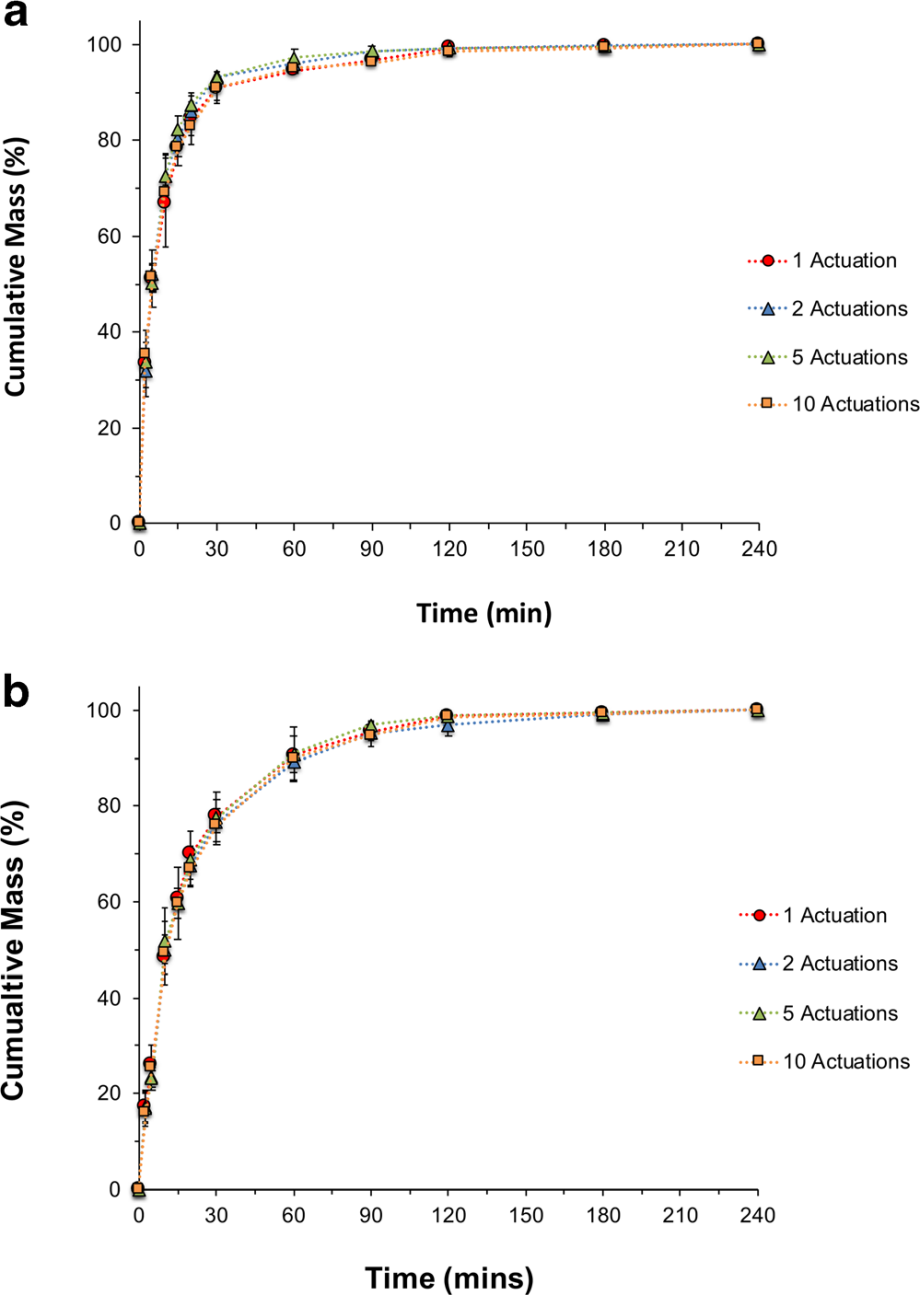
Cumulative mass (%) dissolution profiles of the FP ISM dose with increasing number of actuations of **a** 250 μg FP DPI collected using the ADC system at 60 L/min, and **b** 125 μg FP MDI collected using the ADC system at 30 L/min (n = 3, mean ± SD).

Drug coated filters from the ADC system were carefully loaded and secured onto a stainless steel disk assembly for Paddle over Disk (POD) for dissolution measurements. The dissolution release profiles, plotted as cumulative mass percentage (%) of the total dose recovered after 4 h, of FP with increasing number of actuations (1, 2, 5, and 10) from a commercial 250 μg FP DPI and a 125 μg FP MDI, are shown in Fig. 5a and b, respectively. These plots suggest that the dissolution release profiles of FP were independent of drug loading (approximately 50-500 μg) even though the surface coverage of FP on the filters varied, on average, between 2.6 and 38.3 μg/cm^2^. These findings are supported by similarity factor (f2) analysis of the dissolution profiles. The f2 values between all the different numbers of actuations were between 84–85 and 86–88 for the DPI and MDI dissolution profiles, respectively.

In comparing the dissolution release profiles from the 250 μg FP DPI and 125 μg FP MDI products, the dissolution rate of the ISM dose of FP appeared to be significantly faster from the DPI than the MDI. These observations were supported by the f2 analysis (f2 = 35), mean dissolution time (MDT), and first order release modeling results of the profiles, which are summarized together with the data from other product strength in Table I. While these findings could be related to differences in the upper aerodynamic cut-off diameters of the collected ISM dose for the MDI (≤ 11.7 μm) and the DPI (≤ 8.1 μm), there appears a link with observed *in vivo* differences in the mean absorption time of FP from MDI and DPI products (12). Thorsson *et al.* (2001) indicated that the rate of absorption of FP upon inhalation in healthy patients was slower from the MDI than the DPI (13). These findings are further supported by recent animal testing by Kuehl *et al.* who indicated a marked decrease in the systemic absorption rates of FP from a commercial MDI product with respect to dry powder formulation preparations (14).

**Table I:** Drug loading and dissolution kinetics of the FP ISM dose (n = 3, mean ± SD) for different portable inhaler devices and their different product strengths.

Product	Labeled Dose (μg)	FP Loading (%*w*/w)	ISM (μg)	k_1_ (min^−1^)	T_0.5_ (min)	MDT (min)
FP MDI	50	0.08	75.2 ± 5.9	0.060 ± 0.003	11.64 ± 1.10	20.80 ± 1.64
	125	0.13	118.0 ± 6.4	0.064 ± 0.003	10.78 ± 0.84	19.41 ± 1.02
	250	0.32	99.2 ± 8.5	0.061 ± 0.003	11.45 ± 0.60	19.40 ± 0.58
FP DPI	100	0.79	109 ± 4.9	0.110 ± 0.001	6.32 ± 0.12	8.83 ± 0.60
	250	1.96	97.4 ± 7.2	0.097 ± 0.003	7.15 ± 0.28	9.85 ± 0.83
	500	3.85	97.2 ± 8.6	0.092 ± 0.006	8.13 ± 0.42	11.07 ± 0.91
S/FP DPI	50/100	0.79	110.3 ± 2.9	0.138 ± 0.03	5.03 ± 0.17	6.30 ± 0.49
	50/250	1.95	108.6 ± 1.9	0.112 ± 0.006	6.18 ± 0.43	9.87 ± 0.80
	50/500	3.83	109.4 ± 4.8	0.106 ± 0.008	7.35 ± 0.61	10.49 ± 1.03

*k*_*1*_ First order rate constant, *T*_*0.5*_ The mean dissolution half-life of the drug release, *MDT* Model independent mean dissolution time

The increase in dissolution rate of FP from an interactive mixture is supported by a large body of evidence in solid dosage forms literature (12,15–17). A significant increase in dissolution rate of low solubility, micronized drugs can be achieved when formulated as an interactive mixture with a soluble excipient (12,15–17). These studies indicate that the deagglomeration and distribution of discrete fine particles over the surface of a soluble carrier particle can lead to dissolution rates that are even higher than that of a well dispersed suspension of a micronized drug. The pre-requisite for these high rates of dissolution is directly related to the instantaneous dissolution of the fine carrier particles (15). It could be argued that the aerosolization and dispersion of the drug from a coarse carrier may lead to fully deaggregated drug particles. However, as indicated by the SEM micrograph in the supplementary information (Fig. S2), a proportion of the collected FP remains dispersed over the surface of fine lactose particles. The rapid dissolution of these fine excipient particles within the lining fluid of the lungs may therefore lead to a significant increase in the surface area of the FP particles available to be wetted by the dissolution media. Furthermore, studies have shown that the addition of fine lactose as a ternary additive with a coarse lactose carrier can lead to a further increase in the dissolution rate of drugs when formulated as a low-dose interactive mixture for oral drug delivery (18,19).

### Relationship Between Dissolution Rate Measurements and *In Vivo* Mean Absorption Time

Mechanistic-based predictions of drug absorption and plasma concentration profiles of low solubility, lipophilic inhaled corticosteroids have suggested that deposition patterns and pulmonary dissolution is the rate-limiting step for local and systemic absorption of these permeable drugs (5,7). In these models, the rate of dissolution is simulated based on solubility measurements and regional deposition patterns from particle size distribution measurements. While these simulated dissolution rates have been shown to correlate well with PK-based measurements of mean absorption time (MAT), attempts to confirm this relationship using *in vitro*-based dissolution measurements have generally failed.

**Fig. 6:**
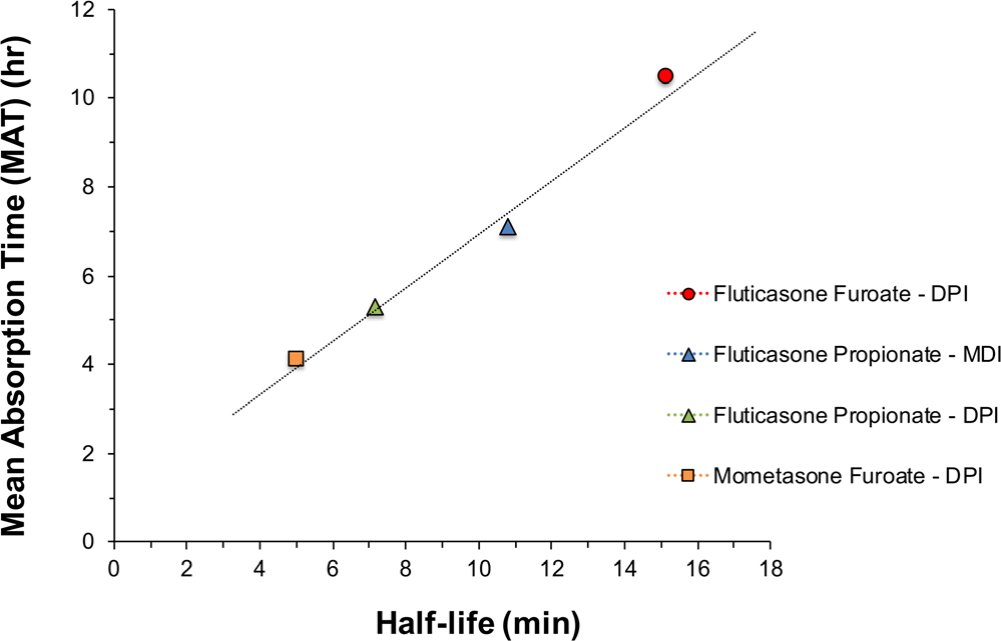
Plot of the mean absorption time (MAT) (17) versus the first order dissolution half-life (n = 3) (experimental data from this work) for a series of inhaled corticosteroids

To support these *in silico* models, the aerosol dissolution of a range of ICS MDI and DPI drug products was compared to the mean absorption time (MAT) of PK measurements from elsewhere (13,20,21). A plot of the literature values of MAT versus dissolution half-life (experimental data from this study) is shown in Fig. 6. These data and the dissolution kinetics are summarized in Table II. *In vitro* dissolution data from this study correlated well with MAT measurements, in the sense that the rate of pulmonary absorption of low soluble, highly permeable ICS molecules is limited by dissolution. Solubility measurements are rather limited as they do not consider physicochemical differences in relation to particle size, surface area, and the actual process of dispersion and deaggregation of the API via an inhaler device. These effects are highlighted by the differences in the FP DPI and MDI formulations, where only dissolution related studies can characterize the direct impact of formulation/device dependency on the dissolution behavior of these compounds.

**Table II:** Mean absorption time (MAT) [16] and dissolution kinetics (calculated using the experimental data from this work, n = 3, mean ± SD) of low aqueous solubility inhaled corticosteroids.

Product	Labeled Dose (μg)	MAT (h)	k1 (min-1)	T0.5 (min)	MDT (min)
FF/V DPI	200/25	10.5	0.046 ± 0.002	15.14 ± 0.98	23.81 ± 2.86
FP MDI	125	7.1	0.064 ± 0.003	10.78 ± 0.84	19.41 ± 1.02
FP DPI	250	5.3	0.097 ± 0.003	7.15 ± 0.28	9.85 ± 0.83
MF DPI	220	4.1	0.138 ± 0.021	4.99 ± 0.74	6.57 ± 1.17

### Influence of Product Strength on *In Vitro* Dissolution of FP

**Fig. 7:**
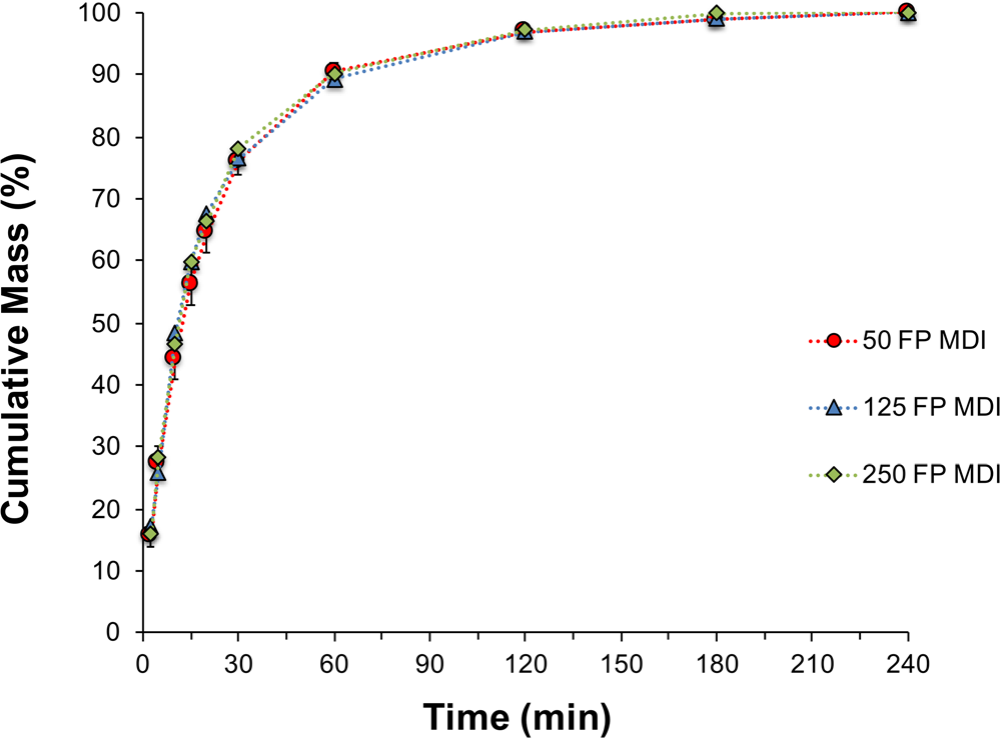
Cumulative mass % dissolution profiles of FP for 1 actuation of the 250 μg, 2 actuations of the 125 μg and 5 actuations of the 50 μg FP MDI collected at a flow rate of 30 L/min (n = 3, mean ± SD)

To investigate the possible influence of different product strengths on the aerosol dissolution properties of micronized fluticasone propionate, dissolution release profiles of commercial 50, 125, and 250 μg FP MDIs, 100, 250, and 500 μg FP DPIs, and 50/100, 50/250, 50/500 μg S/FP DPIs were measured. The dissolution profiles of the ISM dose collected from the three product strengths of FP MDI are shown in Fig. 7. The dissolution kinetics and percentage concentration of FP (% *w*/w) within the MDI products are summarized in Table I. The profiles indicate that the dissolution kinetics of FP was independent of the percentage concentration of the drug within the MDI formulation.

**Fig. 8:**
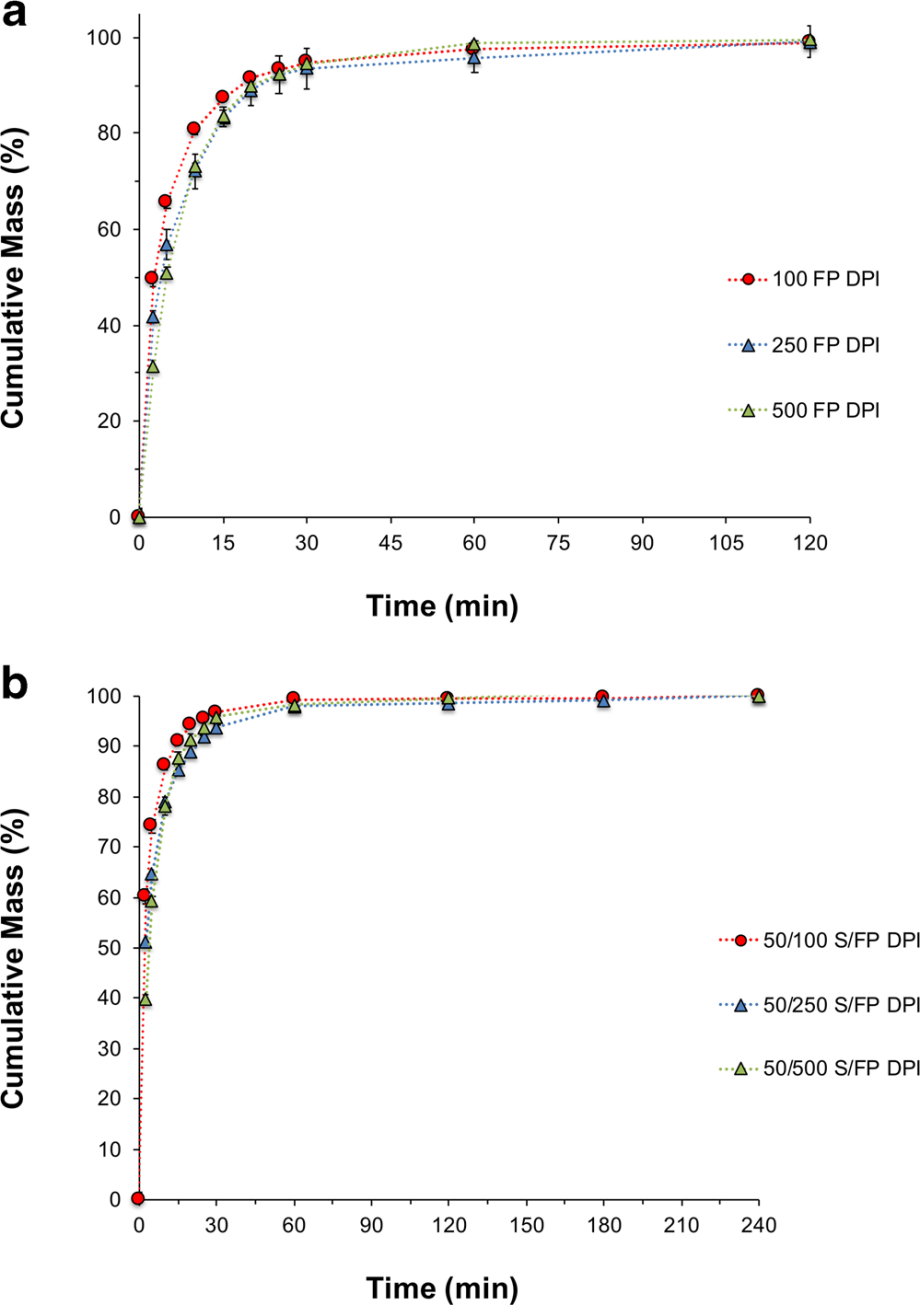
**a** Cumulative mass % dissolution profiles of FP for an equivalent label claim dose of the 100, 250, and 500 μg FP DPIs **b** Cumulative mass % dissolution profiles of FP for an equivalent label claim dose of the 50/100, 50/250, and 50/50 μg S/FP DPIs. Flow rate was set to 60 L/min (n = 3, mean ± SD)

The dissolution profiles, for an equivalent nominal-labeled dose (equivalent to a 500 μg label claim dose), of the FP DPI and S/FP DPI products formulated at three different strengths are shown in Fig. 8a and b, respectively. The dissolution kinetics of the different product strengths are summarized in Table I.

For a fixed concentration of salmeterol (S) and a constant carrier fill weight (12.5 mg), increasing the surface coverage of FP led to a concomitant decrease in rate of dissolution in both mono- and dual-therapy combination products (22). Similarity factor (f2) analysis suggested that none of the product strengths, for both FP DPI and S/FP DPI, had a similar dissolution profile. The decrease in rate of dissolution with increasing drug concentration is supported by previous studies that have shown that the dissolution rate of a poorly soluble compound in an interactive mixture is inversely proportional to the degree of surface coverage and more specifically to the surface area ratios between drug and carrier (15,18). These studies suggested that with increasing drug loading, there is a greater likelihood of drug–drug agglomerate formation over discrete drug particle–carrier interactions.

Interestingly, the presence of a fixed dose of 50 μg micronized salmeterol (S) within the carrier blends led to an increase in the rate of dissolution of the FP in the S/FP DPI products. These observations are supported by f2 similarity factor analysis, which indicated that the dissolution profiles of FP from the collected ISM dose of the 100, 250, and 500 μg FP DPIs were dissimilar to the respective concentrations of FP in the S/FP DPI products. The increase in the rate of dissolution of FP within the dual-therapy combination products may suggest that more soluble salmeterol could play a supporting role in facilitating the increase in area of exposure of the FP, particularly due to the high solubility of the salmeterol and lactose within the dissolution media.

## CONCLUSIONS

In this study, we have designed and engineered a novel dose collection system for *in vitro* dissolution testing of orally inhaled drug products that uniformly distributed the whole impactor stage mass (ISM) onto a single membrane surface. The validated dose collection method was utilized to demonstrate that dissolution profiles of both commercial MDI and DPI products were independent of loaded dose over a wide range of concentrations of drug loading. The independence of the dissolution rate measurements with loaded mass allowed quantitative comparisons to be made between formulation characteristics and dissolution behavior. The increase in robustness and the discriminatory capability of the dissolution method developed in this work may enable quantitative-based comparisons of orally inhaled drug products (inter- and intra-batches) and may aid in the development of a standardized dissolution method for compendial testing of orally inhaled drug products.

## Electronic supplementary material

ESM 1 Visualization of the uniformity in deposition of an ISM dose using the ADC system, with increasing number of actuations (1, 2, 5, and 10) of an alcoholic ink formulated as a 0.5% *w*/*v* solution-based MDI. Flow rate was set to 30 L/min. (PNG 2516 kb)

High resolution image (TIFF 74474 kb)

ESM 2 Scanning electron microscope (SEM) micrograph of the microstructure of the aerosolized ISM dose collected using the ADC system from the 250 μg FP DPI at 60 L/min. The coarser particles appear to be fine particles of lactose. Magnification × 3500 (PNG 2360 kb)

High resolution image (TIFF 56860 kb)
