# Supplementary figures and images for: Development of an Aerosol Dose Collection Apparatus for In Vitro Dissolution Measurements of Orally Inhaled Drug Products
Source: AAPS J. 2020 Feb 13;22(2):47. doi: 10.1208/s12248-020-0422-y (PMC7021740; doi:10.1208/s12248-020-0422-y)

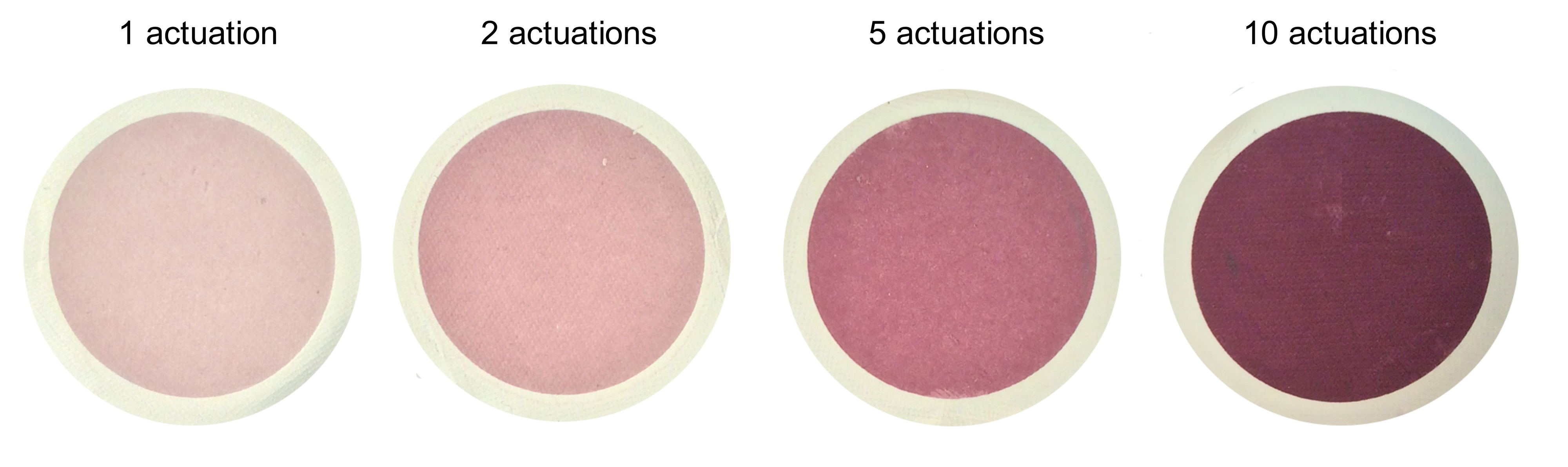

Supplement: Supplementary file 1 — Visualization of the uniformity in deposition of an ISM dose using the ADC system, with increasing number of actuations (1, 2, 5, and 10) of an alcoholic ink formulated as a 0.5% w/v solution-based MDI. Flow rate was set to 30 L/min. (PNG 2516 kb) [file 12248_2020_422_Fig9_ESM.png]

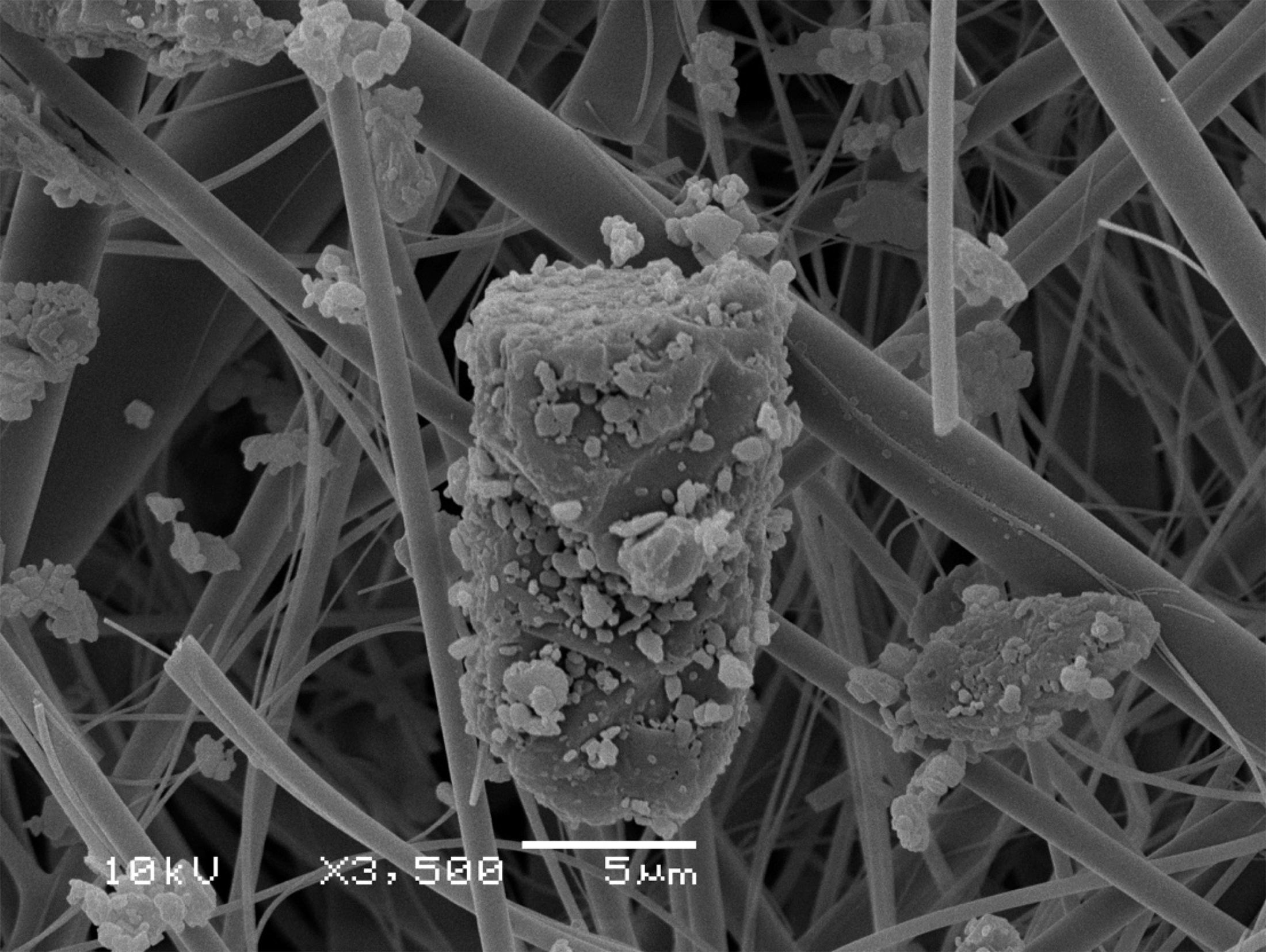

Supplement: Supplementary file 3 — Scanning electron microscope (SEM) micrograph of the microstructure of the aerosolized ISM dose collected using the ADC system from the 250 μg FP DPI at 60 L/min. The coarser particles appear to be fine particles of lactose. Magnification × 3500 (PNG 2360 kb) [file 12248_2020_422_Fig10_ESM.png]

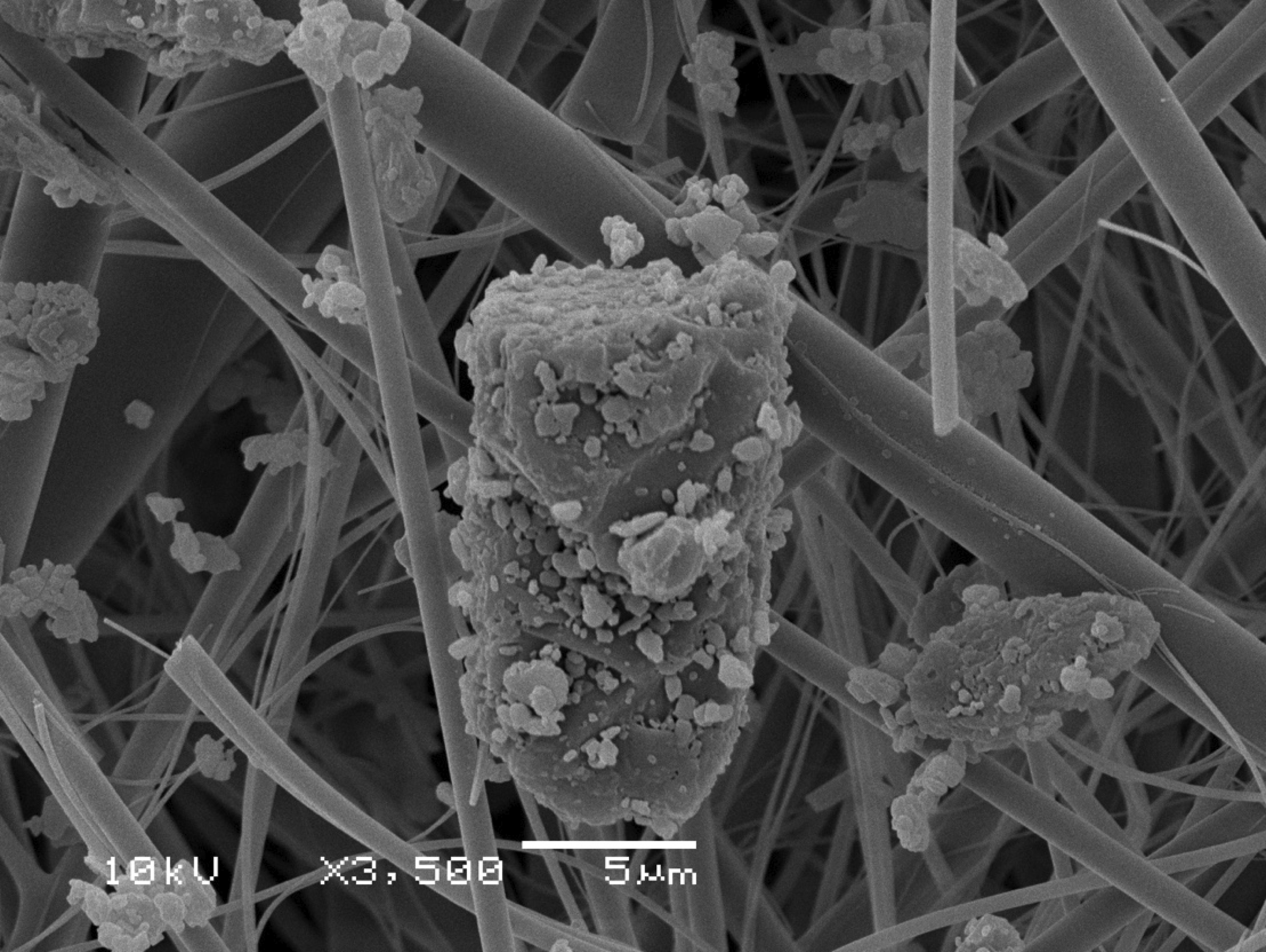

Supplement: Supplementary file 4 — High resolution image (TIFF 56860 kb) [file 12248_2020_422_MOESM2_ESM.tiff]
